# Exploring the Application of Gold‐Assisted Exfoliation in Large‐scale Integration of n‐Type and p‐Type 2D‐FETs

**DOI:** 10.1002/smtd.202500559

**Published:** 2025-07-16

**Authors:** Małgorzata Giza, Krishnendu Mukhopadhyay, Harikrishnan Ravichandran, Andrew Pannone, Subir Ghosh, Saptarshi Das

**Affiliations:** ^1^ Engineering Science and Mechanics Penn State University University Park State College PA 16802 USA; ^2^ Faculty of Physics Warsaw University of Technology Koszykowa 75 Warsaw 00–662 Poland; ^3^ 2D Crystal Consortium Materials Innovation Platform Penn State University University Park State College PA 16802 USA; ^4^ Materials Science and Engineering Penn State University University Park State College PA 16802 USA; ^5^ Electrical Engineering Penn State University University Park State College PA 16802 USA; ^6^ Materials Research Institute Penn State University University Park State College PA 16802 USA

**Keywords:** CMOS inverter, field‐effect transistor, gold‐assisted mechanical exfoliation, large‐area monolayers, transition metal dichalcogenides

## Abstract

2D materials, with their atomic‐scale thickness and exceptional electronic properties, hold immense potential for advancing transistor technologies beyond silicon's limitations. While large‐area growth techniques like metal‐organic chemical vapor deposition (MOCVD) enable scalable device fabrication, achieving monolayers with high crystallinity remains challenging. Recently, gold‐assisted mechanical exfoliation has emerged as a promising alternative, offering large‐area monolayers isolated directly from bulk crystals. In this work, gold‐assisted mechanical exfoliation is utilized to obtain large‐area monolayers of MoS_2_ and WSe_2_ and fabricate over 100 NMOS and 100 PMOS FETs – the largest statistical dataset of FETs created with gold‐assisted exfoliation and the first to include p‐FET performance analysis. Leveraging these devices, the performance of CMOS inverter circuits is constructed and evaluated. This study establishes gold‐assisted exfoliation as a reliable technique for obtaining large‐area 2D materials and highlights the need to optimize bulk crystal growth processes for large‐area monolayer production.

## Graphical abstract

This work demonstrates gold‐assisted mechanical exfoliation as an effective method for obtaining large‐area monolayers of MoS_2_ and WSe_2_. Over 200 FETs are fabricated, providing the largest statistical dataset for devices produced by this technique and the first including p‐FETs. The fabricated devices enabled CMOS inverter construction, confirming the technique's reliability and scalability for 2D electronics.

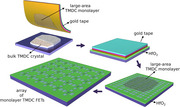

## 1 Introduction

Single‐crystal silicon is foundational to modern electronics due to its high carrier mobility and low defect density, which have enabled the development of reliable and highly miniaturized transistors. Over time, semiconductor manufacturing infrastructure has developed around silicon, further strengthening its dominance in modern electronic technologies. However, further miniaturization requires channel thickness scaling down to sub‐1 nm, where silicon suffers from performance degradation due to increased charge‐carrier scattering at the channel‐dielectric interface.^[^ ^1^, ^2^ ^]^ 2D materials offer a solution by maintaining high carrier mobility at atomic thickness, providing better electrostatic control, and potentially reducing power consumption.^[^ ^3^, ^4^ ^]^ Moreover, recent progress in vertically stacking 2D FETs highlights the ground breaking potential of these materials.^[^ ^5^, ^6^, ^7^ ^]^ Initially, 2D material research relied on mechanical exfoliation, which produces flakes of small lateral dimensions (less than 10 µm) at arbitrary locations.^[^ ^8^ ^]^ This practice prevents the fabrication of a statistically significant number of devices, driving efforts toward large‐area growth. However, scalable synthesis of defect‐free, single‐crystal 2D materials remains challenging; current methods like chemical vapor deposition (CVD) and metal‐organic CVD (MOCVD) often yield polycrystalline films with grain boundaries and lateral sizes ranging from nanometers to single micrometers,^[^ ^9^, ^10^, ^11^ ^]^ compromising electronic properties and uniformity across wafers. Despite significant advancements in growth techniques, as demonstrated by recent reports^[^ ^12^, ^13^, ^14^, ^15^, ^16^ ^]^ showing the ability to grow high‐quality single‐crystal films, challenges such as the process complexity and scalability persist. Such limitations have motivated the exploration of alternative methods for preparing high‐quality 2D materials.

Gold‐assisted mechanical exfoliation has recently gained significant attention as an innovative method for balancing production scalability with the need for high‐quality thin films suitable for electronic applications.^[^ ^17^, ^18^, ^19^ ^]^ This approach is particularly effective for producing single layers of transition metal dichalcogenides (TMDs), utilizing the strong interactions between gold atoms and chalcogens,^[^ ^20^ ^]^ which enables the isolation of large‐area monolayers directly from a bulk crystal. By addressing the scalability limitations of traditional exfoliation techniques while preserving the high crystallinity of monolayers, this method opens new paths for advanced device fabrication. Although the structural properties of monolayers obtained through gold‐assisted exfoliation have shown significant promise,^[^ ^18^, ^21^, ^22^ ^]^ their application in high‐performance electronic devices requires further investigation. The potential of these monolayers for use in field‐effect transistors (FETs) has yet to be fully realized. In particular, developing an understanding of their suitability for not only n‐type but also p‐type devices is necessary for adoption in complementary metal‐oxide‐semiconductor (CMOS) technology.^[^ ^5^, ^6^ ^]^ Addressing these gaps in knowledge is critical for establishing the viability of this fabrication approach in practical electronic applications.

In this study, we utilize gold‐assisted mechanical exfoliation to obtain monolayers of MoS_2_ and WSe_2_, fabricating over 100 n‐FETs and 100 p‐FETs. The large area of the obtained monolayers (9 mm^2^ for MoS_2_ and 5 mm^2^ for WSe_2_) enables the measurement and evaluation of the largest dataset of devices ever reported for both materials, fabricated using this method. While previous studies have investigated n‐type FETs based on monolayer MoS_2_ obtained using this technique, this study provides the first comprehensive analysis of p‐FETs based on monolayer WSe_2_ prepared using this method. Through detailed characterization, we demonstrate consistent field‐effect transistor performance and analyze 50 CMOS inverters created using the fabricated n‐FET and p‐FET devices. Furthermore, we assess the gold‐assisted exfoliation method not only in terms of its ability to produce functional devices, but also its potential scalability for large‐scale production. To achieve this, we identify critical areas for optimization that are essential to facilitate the transition of gold‐assisted exfoliation from a laboratory‐scale method to a scalable solution for industrial applications.

## 2 Results and Discussion

Recently, several exfoliation methods have been reported that leverage the high binding energy between gold and TMDs to isolate TMD monolayers from bulk crystals. These methods are based on either direct exfoliation onto a substrate coated with a gold layer^[^ ^18^, ^23^ ^]^ or the use of gold tape to separate layers from the bulk crystal.^[^ ^19^, ^21^, ^24^ ^]^ For applications involving the fabrication of FETs, the gold tape‐based method proves to be more advantageous, as it allows the exfoliated monolayer to be transferred directly from gold tape onto any desired substrate. Accordingly, we utilized a TRT/PMMA/Au stack, hereafter referred to as gold tape, for the exfoliation of TMD monolayers in this study. The details of the gold tape fabrication process are provided in the Methods Section.

**Figure 1:**
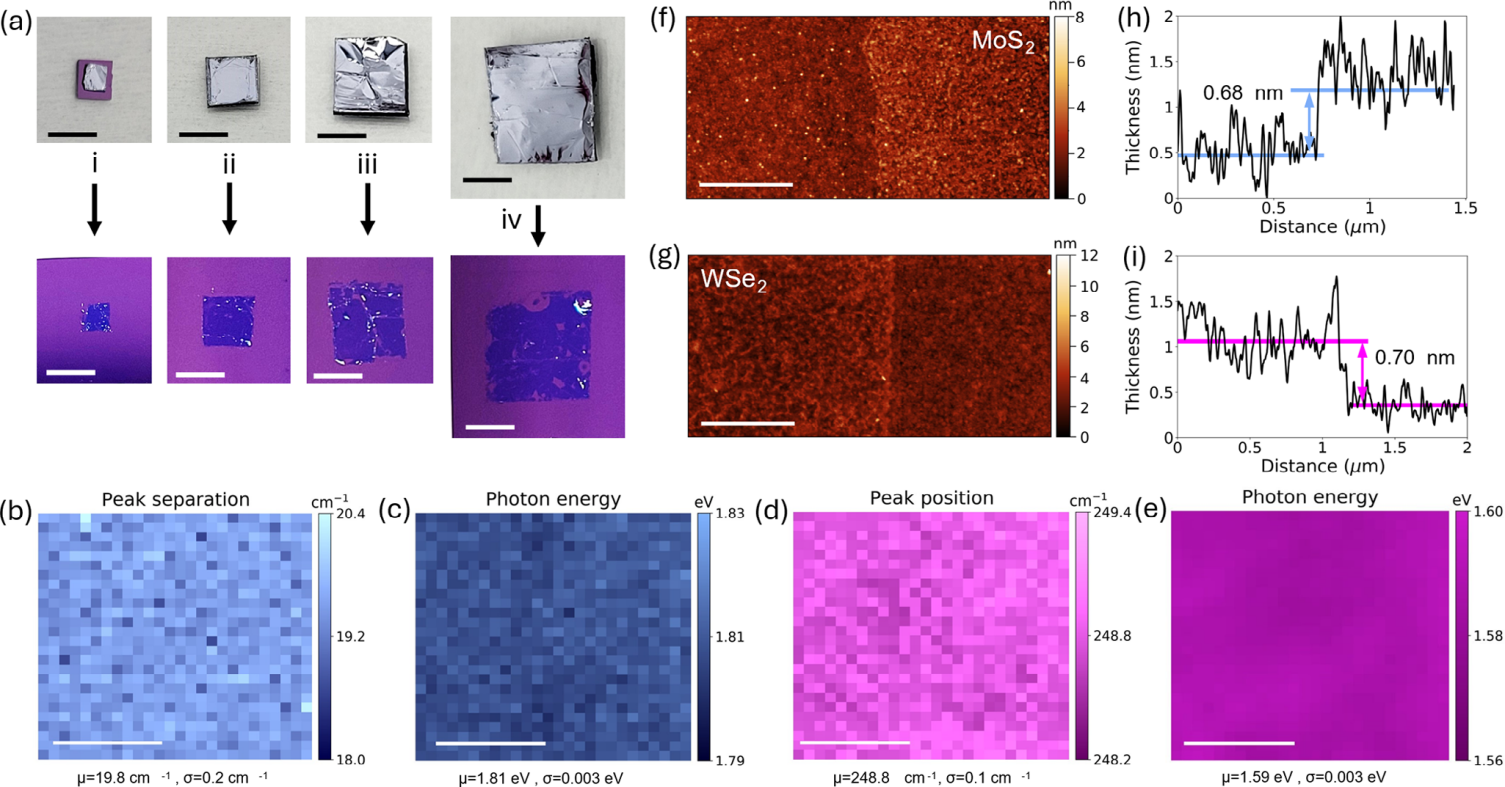
Characterization of monolayer MoS_2_ and WSe_2_ films exfoliated on SiO_2_/Si substrates. a) Optical images of four MoS_2_ bulk crystals with varying sizes and flatness and the corresponding monolayers produced from these bulk crystals on SiO_2_/Si substrates using gold‐assisted exfoliation. White spots on the monolayers represent multilayered flakes exfoliated along with the monolayers. Scale bars are 0.4 cm. b) Raman map of MoS_2_ *A*_1*g*_ and $E_{2g}^{1}$ peak separation. c) PL map of MoS_2_ A exciton energy. d) Raman map of combined WSe_2_ $E_{2g}^{1}$ and *A*_1*g*_ peak position. e) PL map of WSe_2_ A exciton energy. Scale bars in Raman and PL maps are 40 µm. AFM image of f) MoS_2_ monolayer and g) WSe_2_ monolayer on the SiO_2_/Si substrate. Scale bars on AFM images are 500 nm. Thickness profile of h) MoS_2_ monolayer and i) WSe_2_ monolayer extracted from the corresponding AFM image.

The size and continuity of the monolayers obtained using gold tape are primarily determined by a few factors. **Figure** **1**a presents the optical images of MoS_2_ parent bulk crystals [**i‐iv**] and corresponding monolayers exfoliated from those crystals on SiO_2_/Si substrates. Generally, larger bulk crystals yield larger monolayer areas; however, the exfoliated layers are not always continuous. Moreover, in certain regions, multilayers are present, which is attributed to the non‐ideal flatness of the parent bulk crystal. Among the samples studied, the best results were achieved with crystal ii, visually identified as the flattest of the four examined crystals. Achieving continuous, uniform, large‐area monolayers, essential for scalable production of 2D material‐based devices, requires bulk crystals of uniform surface and sufficient size. While growing uniform high‐quality layered crystals remains a challenge, these observations provide a useful perspective for refining exfoliation and bulk crystal purity. As demonstrated in previous studies^[^ ^25^ ^]^ even natural MoS_2_ crystals can contain various impurities, potentially responsible for degrading material properties such as charge carrier mobility. This highlights the importance of not only optimizing the exfoliation process but also carefully controlling the purity of the bulk crystals used as starting materials for device fabrication.

In our study, to further investigate the uniformity and confirm the successful exfoliation of monolayers, Raman spectroscopy and photoluminescence (PL) mapping were performed on MoS_2_ and WSe_2_ monolayers exfoliated on SiO_2_/Si substrates. Each map was acquired over an area of 100 µm × 100 µm. Figure 1b presents a map showing the separation between the two primary Raman active modes found in MoS_2_. The separation between the $E_{2g}^{1}$ and A_1g_ peaks ranges from 18.6 to 20.4 cm⁻¹ across the entire area, confirming the presence of a monolayer of MoS_2_ throughout the region.^[^ ^26^, ^27^, ^28^ ^]^ Figure S1 (Supporting Information) includes a correlative plot of the $E_{2g}^{1}$ and A_1g_ peak positions, which highlights local variations in the vibrational modes and indicates the presence of strain within the MoS_2_ monolayer. Although the observed strain is relatively small, its magnitude is consistent with previous studies on monolayers exfoliated using gold.^[^ ^19^ ^]^ Additional indications of strain are seen in the positions of the MoS_2_ A exciton peak, presented in Figure 1c, which are slightly shifted to lower energies compared to those of a free‐standing MoS_2_ monolayer.^[^ ^29^ ^]^ The narrow distribution, ranging from 1.80 to 1.82 eV, reflects the material's uniformity over the studied area, with minimal variation in the monolayer's structure and composition – an essential factor for reliable device performance. For WSe_2_, Figure 1d presents the position map of the near‐degenerate $E_{2g}^{1}$ and A_1g_ peaks, which remain relatively constant across the mapped region, confirming the homogeneity of the WSe_2_ film. Figure S2a (Supporting Information) includes Raman spectra of monolayer WSe_2_, where the absence of the B_2g_ peak, which is associated with interlayer interactions,^[^ ^30^ ^]^ verifies the successful isolation of the monolayer. Figure 1e shows the energy of the WSe_2_ A exciton peak with an average value of ≈1.59 eV. This value is slightly lower than previously reported for unstrained WSe_2_,^[^ ^31^ ^]^ indicating the presence of strain within the monolayer. A representative Raman spectrum of MoS_2_, along with representative PL spectra of WSe_2_ and MoS_2_ is provided in Supporting Information 2. Atomic force microscopy (AFM) maps measured across a 2 µm x 1 µm region are depicted in Figure 1f,g, and extracted line‐scans shown in Figure 1h,i reveal that the obtained films are monolayers with thicknesses of ≈0.68 and 0.70 nm for MoS_2_ and WSe_2_, respectively.

**Figure 2:**
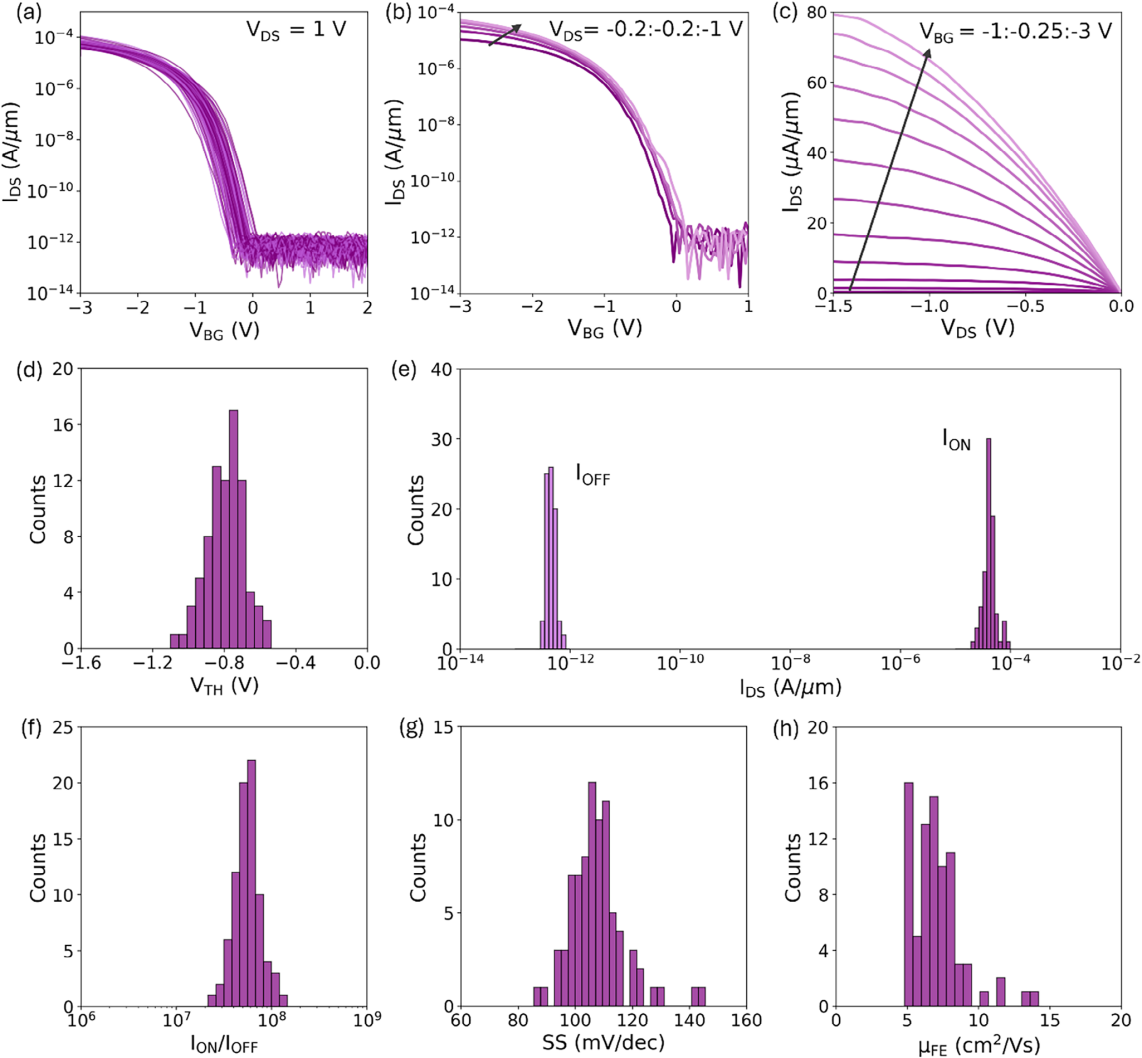
Characterization of monolayer WSe_2_ p‐FETs. Transfer characteristics of a) 120 WSe_2_ p‐FET devices measured at a *V_DS_* of 1 V and b) a representative p‐FET device measured with *V_DS_* ranging from −0.2 to −1 V with −0.2 V step. c) Output characteristics of representative p‐FET measured with *V_BG_* varying from −1 to −3 V with a step of −0.25 V. Distribution of d) threshold voltage *V_TH_*, e) current in on‐state and off‐state operation of the devices, f) *I_ON_*/*I_OFF_* ratio, g) subthreshold slope (SS) and h) field‐effect mobility (µ_*FE*_).

For device fabrication, monolayers of MoS_2_ and WSe_2_ were exfoliated with the previously described gold‐assisted method on separate substrates with individually accessible local back‐gate electrodes, where 10 nm HfO_2_ serves as the gate dielectric. In this study, a single exfoliation was performed for each material. All WSe_2_ FETs were fabricated on a single substrate, and similarly all MoS_2_ FETs were fabricated on a separate single substrate. This approach enables a direct evaluation of device performance and uniformity across large‐area monolayers produced by gold‐assisted exfoliation. Figure S3 (Supporting Information) includes optical images depicting exfoliated monolayers on a substrate and devices fabricated on the same exfoliated monolayers. All devices were designed to have a 200 nm channel length and 600 nm channel width. To fabricate p‐FETs, we deposited 10 nm Pd and 20 nm Pt as contact metals. A detailed description of device fabrication is presented in the Methods Section. **Figure** **2**a presents the transfer characteristics of 120 p‐FET devices measured at a drain voltage (V_DS_) of 1 V, showcasing good uniformity. Figure 2b shows the transfer characteristics of a representative device measured at different V_DS_ values, revealing an increased on‐state current with a higher magnitude of V_DS_. Notably, the off‐state current remained constant and had a value on the same order as the leakage current I_G_ (≈pA µm^−1^ presented in Figure S4a, Supporting Information), highlighting good gate control over the channel. Figure 2c shows output characteristics for the same representative device measured at gate voltages (V_BG_) ranging from −1 to −3 V. The nonlinear relationship between the drain current (I_DS_) and V_DS_ indicates that the device is Schottky limited.

To assess device‐to‐device variation, we calculated key FET parameters based on acquired transfer characteristics. These metrics provide critical insights into the uniformity and performance of the fabricated devices. Figure 2d presents the distribution of threshold voltage (V_TH_) extracted using the constant current method at an iso‐current of 100 nA µm^−1^. The median V_TH_ was −0.8 V with a standard deviation of 0.1 V. These values indicate a relatively narrow distribution of V_TH_, suggesting good uniformity of the monolayers, which is critical for the potential use of gold‐assisted exfoliation in scalable integration of 2D‐material‐based devices. Figure 2e shows the distributions of on‐state (I_ON_) and off‐state (I_OFF_) current. I_ON_ was extracted for an overdrive voltage of 1.9 V, which corresponds to a carrier concentration (n_s_) of 1.8 × 10^13^ cm^−2^. The maximum achieved value of I_ON_ was 88 µA µm^−1^, while the median was 41 µA µm^−1^ with a standard deviation of 12 µA µm^−1^. These results demonstrate the ability of WSe_2_‐based FETs to achieve substantial on‐currents, comparable to values reported in the literature for similar p‐type 2D‐FETs fabricated both with traditional mechanically exfoliated flakes^[^ ^32^, ^33^ ^]^ and large‐area grown films (MBE, MOCVD, and CVD).^[^ ^34^, ^35^, ^36^ ^]^ This comparison emphasizes the viability of gold‐assisted exfoliation as an alternative technique to obtain high‐quality monolayers. I_OFF_ values were determined as the average current during the devices' off‐state operation, with V_BG_ ranging from 1 to 2 V. Figure 2f shows the I_ON_/I_OFF_ ratio distribution. The median value of I_ON_/I_OFF_ ratio is 5.6 × 10^7^, with a standard deviation of 2 × 10^7^. This high I_ON_/I_OFF_ ratio indicates excellent switching behavior, ensuring minimal static power consumption, which further enhances the applicability of these devices in energy‐efficient electronic circuits. Figure 2 g shows the subthreshold slope (SS) distribution, which was calculated over two orders of magnitude of current increase. The lowest observed SS was 85 mV dec^−1^, while the median SS was 107 mV dec^−1^ with a standard deviation of 10 mV dec^−1^. Achieving such low SS values is particularly promising for applications requiring high‐speed and low‐energy transistors. Figure 2h shows the distribution of field‐effect mobility (µ_FE_) calculated using the peak transconductance method. The highest recorded µ_FE_ was 14.2 cm^2^ Vs^−1^, while the median value was 6.8 cm^2^ Vs^−1^ with a standard deviation of 1.8 cm^2^ Vs^−1^. Although the median mobility is moderate, the observed maximum value suggests that further optimization of device interfaces and processing steps could lead to significant enhancements in electrical performance. It is worth noting that the extracted mobility can be underestimated due to the impact of contact resistance, particularly in devices with channel lengths below 1 µm,^[^ ^37^ ^]^ as well as the presence of residual contaminants from the transfer process, which may introduce additional scattering centers and degrade carrier transport.^[^ ^38^ ^]^ Reduced interfacial trap densities and improved contact engineering could help to unlock the full potential of monolayer 2D TMDCs.^[^ ^39^, ^40^ ^]^ Notably, these are the first devices fabricated with monolayer WSe_2_ obtained using gold‐assisted exfoliation, underscoring the novelty of this study. The analysis of device‐to‐device variation reveals a high degree of uniformity and promising performance metrics for monolayer WSe_2_‐based FETs fabricated with this method.

**Figure 3:**
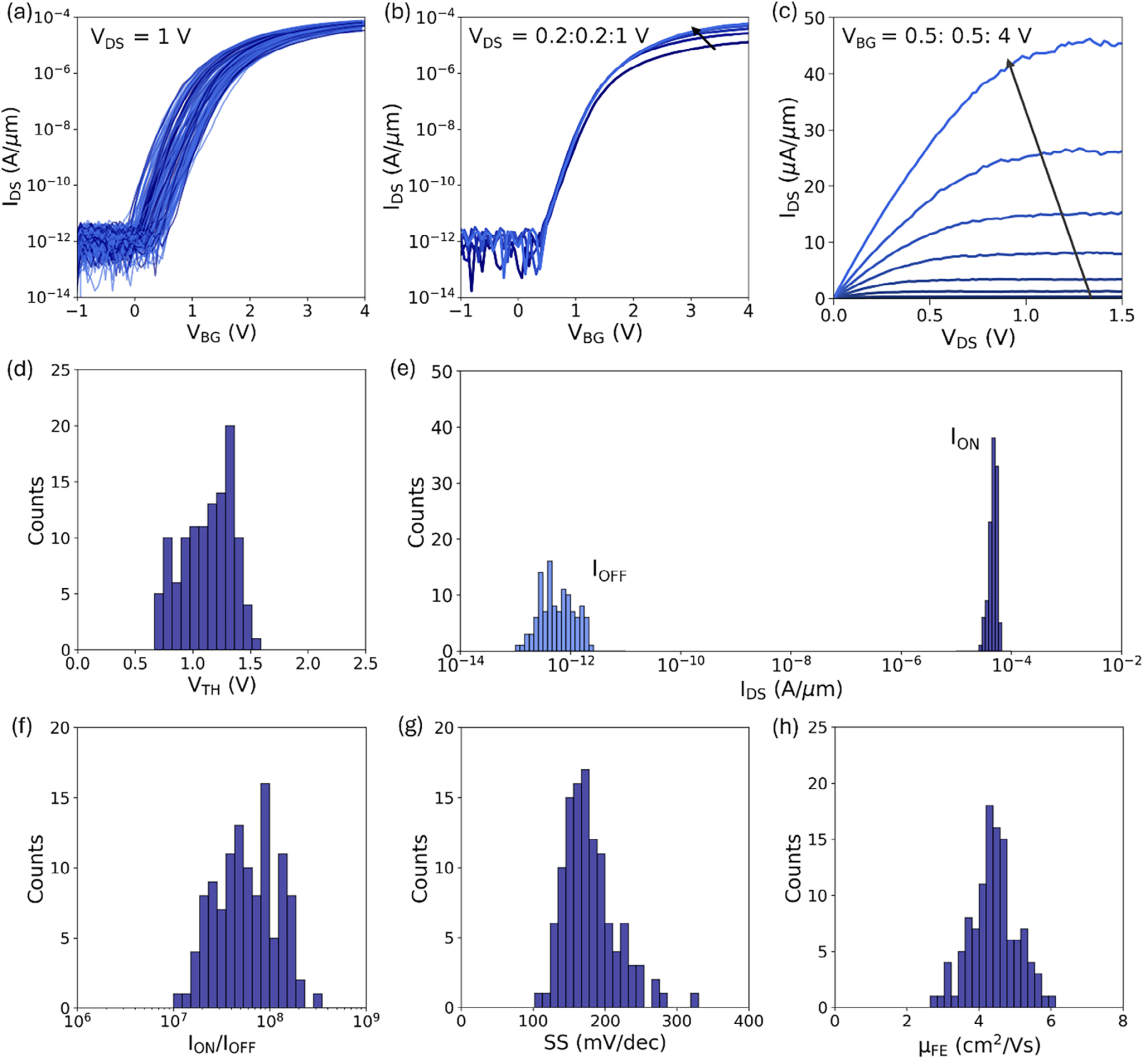
Characterization of monolayer MoS_2_ n‐FETs. Transfer characteristics of a) 120 MoS_2_ n‐FET devices measured at *V_DS_* of 1V and b) a representative n‐FET device measured at *V_DS_* ranging from 0.2 to 1 V with a 0.2 V step. c) Output characteristics of a representative n‐FET measured with *V_BG_* varying from 0.5 to 4 V with a step of 0.5 V. Distribution of d) threshold voltage (*V_TH_*), e) current in on‐state and off‐state operation of the devices, f) *I_ON_*/*I_OFF_* ratio, g) subthreshold slope (SS) and h) field‐effect mobility (µ_*FE*_).

A similar analysis was performed for devices fabricated on MoS_2_ monolayers. To achieve n‐FETs, a stack of 30 nm of Au, 5 nm Ti, and 30 nm of Pt was deposited as contact metals. **Figure** **3**a shows the transfer characteristics of 120 FETs based on MoS_2_ monolayers measured at a V_DS_ of 1 V. The devices exhibit n‐type behavior, demonstrating consistent electrical characteristics across the batch. Figure 3b shows the transfer characteristics of a representative device measured over a range of V_DS_ values from 0.2 to 1 V. In the on‐state, the I_DS_ increased proportionally with higher V_DS_, while the off‐state current remained stable across all applied voltages and had a value on the same order as the I_G_ (≈pA µm^−1^ presented in Figure S4b, Supporting Information). Figure 3c presents output characteristics of the same representative device, measured for V_BG_ ranging from 0.5 to 4 V. The observed nonlinear relationship between I_DS_ and V_DS_ confirms the Schottky‐type contacts in MoS_2_‐based devices. In Figure 3d, we present the distribution of V_TH_ extracted using the constant current method at an iso‐current of 100 nA µm^−1^. The median value of V_TH_ was 1.2 V with a standard deviation of 0.2 V. Although the distribution remains narrow, it is slightly larger than that observed for the V_TH_ of devices fabricated on WSe_2_. This could be attributed to residue remaining on the MoS_2_ sample, as evidenced by the AFM scans shown in Figure 1f, which most likely originates from PMMA, a component of the gold tape used during the exfoliation process. The presence of residue indicates that the gold‐assisted exfoliation method may require optimization for specific 2D TMD materials. Figure 3e presents the distributions of I_ON_ and I_OFF_ current. I_ON_ was calculated for an overdrive voltage of 2.4 V, corresponding to a n_s_ of 2.3 × 10¹^3^ cm⁻^2^. I_OFF_ is extracted as the average I_DS_ value for V_BG_ ranging from −0.5 to −1 V. The highest obtained I_ON_ value was 63 µA µm^−1^, while the median value was 48 µA µm^−1^ with a standard deviation of 7 µA µm^−1^. The obtained values are comparable to those reported in other studies on gold‐assisted exfoliated materials;^[^ ^41^ ^]^ however, the I_ON_ level remains significantly lower than that achieved in transistors fabricated on MoS_2_ monolayers obtained by growth techniques.^[^ ^42^, ^43^ ^]^ The performance comparison suggests that if gold‐assisted mechanical exfoliation is to be considered a viable method for large‐scale device production, further optimization is essential to achieve clean, high‐quality, and uniform layers. Additionally, the quality of the bulk crystal used for exfoliation must also be carefully evaluated, not only in terms of its flatness and size but also with respect to its crystalline purity, which plays a crucial role in achieving high‐performance devices. While the gold‐assisted exfoliation can enable access to large‐area monolayers, the bulk crystals may contain native defects that are difficult to identify and control, potentially affecting the electrical performance of the resulting devices.^[^ ^25^ ^]^ Moreover, the gold‐assisted exfoliation process involves the use of a protective polymer layer such as PMMA, which can introduce surface residues and interfacial contamination. These contaminants not only increase contact resistance but can also impact carrier mobility. Addressing these issues, through refinement of both gold‐assisted exfoliation processes and bulk crystal quality, will be essential for realizing the full potential of gold‐assisted exfoliation in scalable device applications.

While the I_ON_ current in the presented devices is moderate, the obtained distribution of I_ON_/I_OFF_ ratio shown in Figure 3f is high. The maximum I_ON_/I_OFF_ ratio is 3 × 10^8^, while the median value is 5.7 × 10^7^. with a standard deviation of 5.2 × 10^7^. The high I_ON_/I_OFF_ ratios confirm excellent switching behavior, essential for logic device applications, particularly where low leakage and high drive currents are required. The distribution of subthreshold slope (SS) values, calculated for a two‐order magnitude change in I_DS_, is shown in Figure 3g. The lowest SS value achieved for n‐FETs was 102 mV dec^−1^, with a median value of 172 mV dec^−1^ and a standard deviation of 37 mV dec^−1^. Notably, the lowest SS value of 102 mV dec^−1^ is comparable to the only study^[^ ^18^ ^]^ reporting SS values for devices fabricated on MoS_2_ monolayers using the gold‐assisted exfoliation method, highlighting the limited research on this specific fabrication technique. The field‐effect mobility (µ_FE_) distribution shown in Figure 3h reveals a median value of 4.4 cm^2^ Vs^−1^, with a standard deviation of 1.2 cm^2^ Vs^−1^ and a maximum value that reaches 6.1 cm^2^ Vs^−1^. Moderate SS and mobility values indicate room for enhancement through further optimization, such as dielectric engineering, improving the channel‐contact interface, and enhancing the overall cleanliness of the surface of exfoliated MoS_2_.

**Figure 4:**
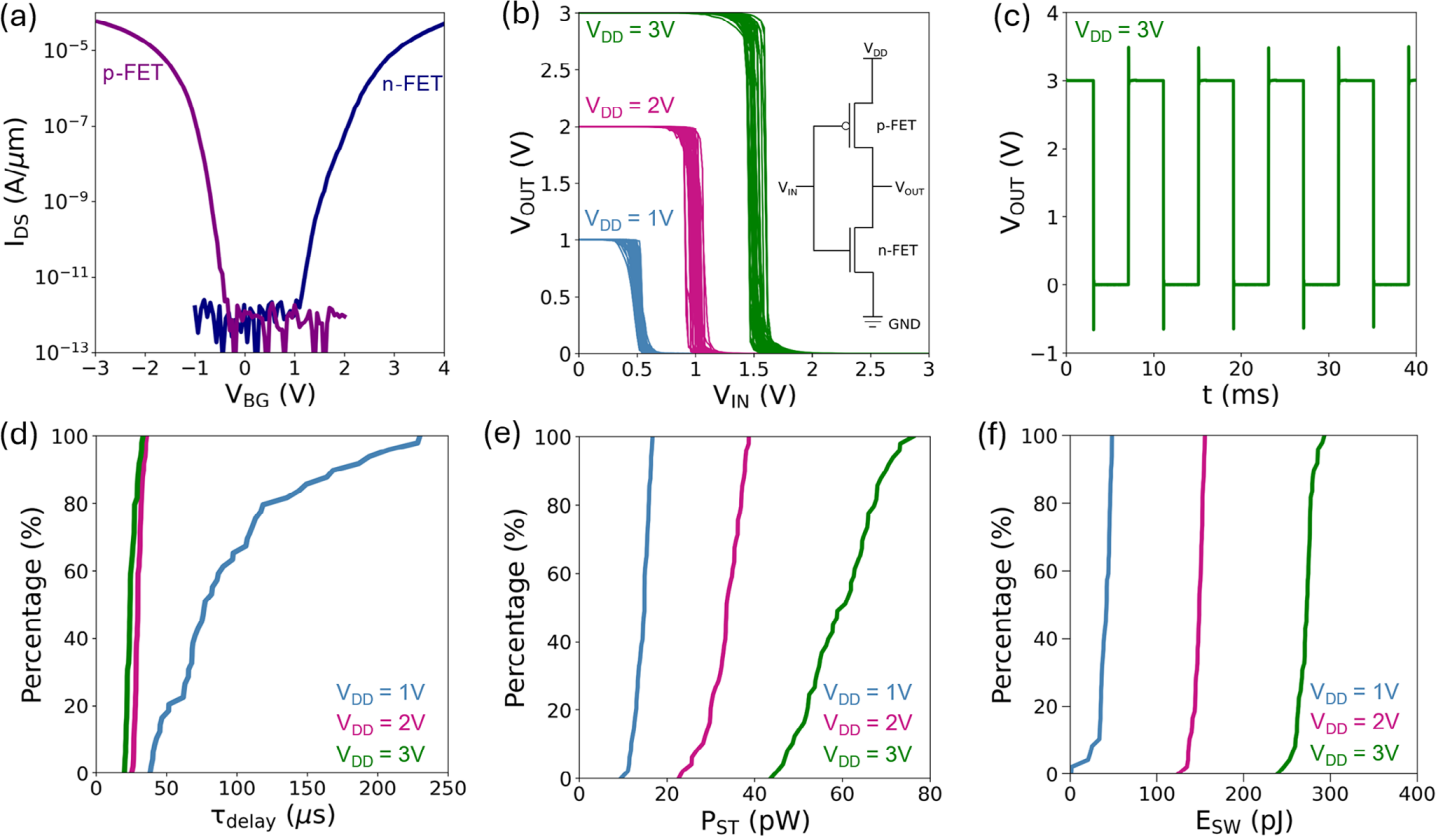
Characterization of 2D CMOS inverters. a) Representative transfer characteristics of a p‐FET and an n‐FET device measured at a *V_DS_* of 1 V. b) Output characteristics of 50 CMOS inverters measured at *V_DD_* of 1, 2, and 3 V. c) Output voltage (*V_OUT_*) of a representative CMOS inverter measured in response to a square‐wave input signal (*V_IN_*) alternating between GND and a *V_DD_* of 3 V. Cumulative distribution function of d) delay time, e) static power and f) switching energy acquired for *V_DD_* = 1, 2 and 3 V.

Benchmarking tables, Table S1 and Table S2, have been included in the Supporting Information to compare key device parameters – such as I_ON_, subthreshold slope, and I_ON_/I_OFF_ current ratio – for MoS_2_ and WSe_2_ devices fabricated using gold‐assisted exfoliation technique with those fabricated by CVD, MBE, MOCVD, and conventional mechanical exfoliation methods. Our results show that in certain cases, the I_ON_ achieved via gold‐assisted exfoliation is comparable to or better than the I_ON_ reported for devices fabricated by CVD or MOCVD. Moreover, the subthreshold slope of our devices is consistently improved relative to many other methods. The I_ON_/I_OFF_ ratio also aligns well with state‐of‐the‐art devices reported in literature. However, we acknowledge that some of the best‐performing CVD‐grown devices still outperform our current results in terms of overall electrical performance. We attribute this to residual interfacial contamination introduced during the exfoliation process. We anticipate that further refinement of the gold‐assisted exfoliation technique – with a particular focus on minimizing such contamination – will substantially improve device performance, potentially rendering it competitive with the highest‐performing devices fabricated via CVD and MBE methods. Building upon these promising results for individual transistors, we next explored their integration into complementary logic circuits. To create CMOS inverters from existing devices, we externally connected pairs of n‐FET and p‐FETs. **Figure** **4**a shows transfer characteristics of a representative pair of devices. In total, 50 CMOS inverters were measured under a supply voltage (V_DD_) of 1, 2, and 3 V. Output characteristics of inverters are presented in Figure 4b, exhibiting switching thresholds at $∼\;V_{\mathrm{DD}}/2$. This expected behavior results from the symmetry in the electrical characteristics of the n‐FET (based on MoS_2_) and the p‐FET (based on WSe_2_) that ensure balanced switching. Although the n‐FETs do not exhibit competitive performance, their on‐current levels are comparable to those of the p‐FETs, enabling the construction of inverters with well‐defined switching thresholds and robust logic‐behavior. To further assess the performance of these inverters, speed measurements were conducted by applying a square‐wave input signal (V_IN_) alternating between GND and a V_DD_ of 1, 2, and 3 V. Figure 4c presents the output voltage waveform of a representative inverter at a V_DD_ of 3 V, highlighting a sharp rise and fall in the output voltage. A single cycle of V_OUT_ is presented in Figure S5 (Supporting Information). The cumulative distribution function of the delay time (τ_delay_), calculated as the sum of rise time and fall time, is presented in Figure 4d. The shortest delay time of 20 µs was acquired at a V_DD_ of 3 V, which corresponds to a switching frequency of 50 kHz, indicative of fast operation and low dynamic losses. The median value of τ_delay_ was 24 µs with a standard deviation of 4 µs, surpassing the performance of previously reported MoS_2_‐based NMOS inverters^[^ ^44^ ^]^ and nanotube‐based PMOS inverters.^[^ ^43^ ^]^ The reduction of τ_delay_ with increasing V_DD_ is an improvement in speed that comes at the expense of higher power consumption. The static power (P_ST_) values are shown in Figure 4e. For V_DD_ of 3 V, the lowest P_ST_ value measured was 44 pW, with a median of 60 pW and a standard deviation of 8 pW, which is lower than the energy consumption observed in other inverters made with nanomaterials,^[^ ^44^, ^45^, ^46^ ^]^ highlighting the superior efficiency of the presented devices. These low P_ST_ values result from the low I_OFF_ (≈pA µm^−1^) of both n‐FET and p‐FET devices, making these inverters highly energy‐efficient in idle states. The increasing trend of P_ST_ with higher V_DD_ highlights the trade‐off between static power consumption and operating voltage. Finally, Figure 4f shows the extracted switching energy (E_SW_), which quantifies the energy required for transitioning between logical states. At a supply voltage of 3 V, the distribution of E_SW_ exhibits a median of 273 pJ, with a standard deviation of 10 pJ and a minimum value of 239 pJ. It was observed that E_SW_ increases with rising V_DD_. While this trend allows for faster operation and reduced delay times, it also leads to lower energy efficiency. Low switching energy combined with minimal static power dissipation positions these inverters as promising candidates for energy‐efficient applications. Future efforts could leverage the capabilities of gold‐assisted exfoliation to integrate NMOS and PMOS devices on the same substrate, eliminating the need for external wiring and enabling fully integrated 2D CMOS logic circuits.^[^ ^5^, ^6^ ^]^

## 3 Conclusion

In conclusion, our study demonstrates the successful gold‐assisted exfoliation of MoS_2_ and WSe_2_ monolayers enabling the development of both n‐FETs and p‐FETs. Notably, this is the first demonstration of p‐type transistors fabricated with monolayers obtained using this method. This comprehensive analysis of over 200 devices and 50 CMOS inverters, the largest statistical dataset of devices fabricated with monolayers obtained via gold‐assisted exfoliation, highlights the potential of this technique for large‐scale device integration. While the inverters demonstrate reliable operation, further focus is needed on enhancing the individual performance of both n‐FET and p‐FET devices. Improving the functionality of these transistors could be achieved by enhancing the crystallinity and uniformity of the bulk crystal and the exfoliated layers. Overall, this study is crucial for evaluating the viability of large‐area exfoliation methods in the production of high‐performance electronics based on 2D materials.

## 4 Experimental Section

### Back‐Gate Stack Fabrication

To define the back‐gate, a 285 nm SiO_2_/Si substrate was spin‐coated with a bilayer resist (EL6 and PMMA A3) at 4000 rpm and baked at 150 and 180 °C, respectively for 90 s each. The pattern for the local‐back gates was created using e‐beam lithography, followed by development in a 1:1 solution of MIBK:IPA for 60 s and rinsing in IPA for 45 s. A back‐gate electrode stack, consisting of 5 nm Ti and 15 nm Pt was deposited using electron beam evaporation in a Temescal FC‐2000 Evaporator System. Lift‐off of the metal and resist layers was performed using acetone for 45 min at 60 °C, followed by rinsing in IPA for 5 min. A 10 nm HfO_2_ layer was then deposited across the entire substrate using atomic layer deposition (ALD). To expose the gate contact pads, another e‐beam lithography step was carried out. The etch pattern was defined by spin‐coating ZEP resist at 2500 rpm for 45 s, baking it at 180 °C for 3 min, followed by e‐beam lithography. Development was carried out in n‐amyl acetate for 3 min at room temperature, followed by rinsing in IPA for 60 s. Reactive ion etching was performed using BCl_3_ in the Plasma‐Therm Versalock 700 system to access the gate pads. The resist was subsequently removed by immersing the sample in PRS 3000 solution for 45 min at 60 °C, followed by a final cleaning step with IPA.

### Gold Tape Fabrication

To enable exfoliation, a tape with a 40 nm‐thick gold layer was fabricated. Silicon wafer, which serves as a substrate, was pretreated with oxygen plasma at 50 W RF power for 30 s using the M4L RF gas plasma system. Next, gold was deposited onto the wafer via electron beam evaporation (Temescal FC‐2000 Evaporator) at a deposition rate of 3 Å s^−1^, with the chamber temperature maintained between 20 and 22 °C. The wafer with a gold layer was spin‐coated with PMMA A6 at 1000 rpm for 45 s and baked at 150 °C for 2 min. Subsequently, the wafer was diced into 1 cm × 1 cm pieces. TRT was applied and gently pressed on the gold layer with PMMA coating creating TRT/PMMA/Au/Si stack. The TRT was then peeled off, transferring the PMMA and gold layers onto the tape. The resulting gold tape (TRT/PMMA/Au) was used for the exfoliation of MoS_2_ and WSe_2_ crystals.

### Gold‐Assisted Exfoliation

Before the exfoliation process, bulk MoS_2_ (from 2D Semiconductors) and WSe_2_ (from HQ Graphene) crystals were affixed to a SiO_2_/Si substrate using double‐sided tape. The top layer of the bulk crystal was peeled off using scotch tape, and the gold tape was immediately applied to the crystal surface. After 30 s, the gold tape was separated from the bulk crystal, isolating monolayers of the material, which were then transferred onto a desired substrate. The substrate, along with the gold tape and exfoliated monolayer, was placed on a hot plate set to 150 °C. The TRT was promptly removed using tweezers, and the substrate was kept on the hot plate for 10 min to enhance adhesion between the monolayer and the substrate. To remove the PMMA layer, the substrate was immersed in Microposit Remover heated to 60 °C for 10 min, followed by acetone at 60 °C for another 10 min, and finally rinsed in IPA at room temperature for 2 min. The gold layer was removed by immersing the substrate in TFA Au etchant (KI/I_2_) at room temperature for 5 min. After etching, the substrate was sequentially rinsed in two solutions (10% IPA in DI water) for 30 s each and in IPA for another 30 s. To eliminate residual contaminants, the samples were cleaned in acetone at room temperature for 12 h. A simplified schematic of the gold‐assisted exfoliation process is shown in the Figure S6 (Supporting Information).

### FET Fabrication

FET fabrication was carried out according to procedures described in our previous works,^[^ ^47^, ^48^, ^49^ ^]^ with minor modifications as detailed below. The exfoliated monolayers were coated with PMMA A6 at 4000 rpm, baked at 180 °C for 2 min, and subsequently patterned using e‐beam lithography. The resist was developed in a 1:1 solution of MIBK:IPA for 60 s, followed by rinsing in IPA for 45 s. To define the MoS_2_ and WSe_2_ channels, SF_6_ and O_2_ reactive ion etching was performed using a Plasma‐Therm Versalock 700 system, followed by resist removal in acetone at room temperature for 12 h. Another e‐beam lithography step with a bilayer resist (EL6 and PMMA A3) was used to pattern the source and drain contacts. Development was performed in a 1:1 solution of MIBK:IPA for 60 s, followed by rinsing in IPA for 45 s. For MoS_2_ devices, 30 nm Au, 5 nm Ti, and 30 nm Pt were deposited as contact metals, while for WSe_2_ devices, 10 nm Pd and 20 nm Pt were used. The lift‐off process was carried out by immersing the samples in acetone heated to 60 °C for 45 min. Finally, the devices underwent an NO annealing step at 300 °C for 30 min to achieve doping in the WSe_2_ channels.

### Raman and Photoluminescence Mapping

Raman and photoluminescence (PL) spectroscopy of MoS_2_ and WSe_2_ monolayers were performed using a Horiba LabRAM HR Evolution confocal Raman microscope with a 532 nm excitation wavelength. The laser power was set to 0.34 mW for Raman measurements and 0.034 mW for PL measurements. The spectra were acquired with a 50x objective lens, using an 1800 lines/mm grating for Raman measurements and a 300 lines/mm grating for PL.

### AFM Measurements

The thickness of the monolayers of MoS_2_ and WSe_2_ was measured using Bruker Dimension Icon Atomic Force Microscope operating in peak‐force tapping mode.

### Electrical Characterization

Electrical characterization of FETs was conducted in a Lake Shore CRX‐VF probe station under atmospheric conditions using a Keysight B1500A parameter analyzer. Speed measurements of CMOS inverters were performed with a PZ2121A high speed unit.

## Conflict of Interest

The authors declare no conflict of interest.

## Author Contributions

M.G., K.M., and S.D. conceived the idea and designed the experiments. M.G. and K. M. exfoliated large‐area monolayers. M.G., K. M., and S.G. fabricated devices. M.G., K.M., and H.R. performed electrical measurements. A.P. performed Raman spectroscopy and PL measurements. M.G., K.M. performed AFM measurements. M.G., K.M. analyzed all the acquired data. M.G., K.M., and S.D. wrote the manuscript. All authors contributed to the preparation of the manuscript.

## Supporting information



Supporting Information

## Data Availability

The data that support the findings of this study are available from the corresponding author upon reasonable request.
