# Supplementary material for: Exploring the Application of Gold‐Assisted Exfoliation in Large‐scale Integration of n‐Type and p‐Type 2D‐FETs
Source: Small Methods. 2025 Jul 16;10(3):2500559. doi: 10.1002/smtd.202500559 (PMC12893270; doi:10.1002/smtd.202500559)
Supplement: Supplementary file 1 — Supporting Information [file SMTD-10-2500559-s001.docx]

Supporting Information
Exploring the application of gold-assisted exfoliation in large-scale integration of n-type and p-type 2D-FETs

*Małgorzata Giza^1,2^, Krishnendu Mukhopadhyay^1^, Harikrishnan Ravichandran^1^, Andrew L Pannone^1^, Subir Ghosh^1^ & Saptarshi Das^1,3,4,5,6*^*

*^1^Engineering Science and Mechanics, Penn State University, University Park, PA 16802, USA*

*^2^Faculty of Physics, Warsaw University of Technology, Koszykowa 75, 00-662 Warsaw, Poland
^3^2D Crystal Consortium Materials Innovation Platform, Penn State University, PA 16802, USA
^4^Materials Science and Engineering, Penn State University, University Park, PA 16802, USA*

*^5^Electrical Engineering, Penn State University, University Park, PA 16802, USA*

*^6^Materials Research Institute, Penn State University, University Park, PA 16802, USA*

*^*^Corresponding author: email –* [*sud70@psu.edu*](mailto:sud70@psu.edu)

**Supporting Information 1: Strain and doping analysis in monolayer MoS_2_**


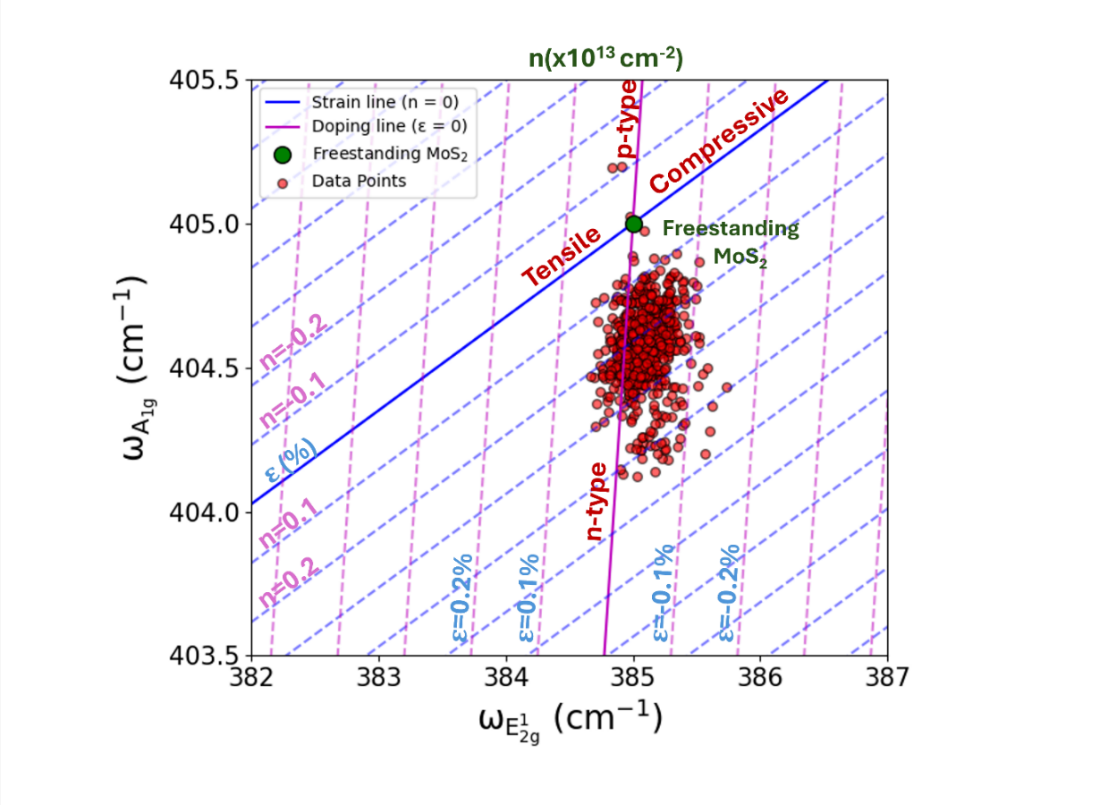


***Figure S1. Strain and doping analysis in monolayer MoS_2_.*** *Correlative plot of the* $E_{2g}^{1}$ *and* $A_{1g}$ *peak positions for evaluating strain and charge doping distributions in a monolayer MoS_2_ exfoliated using the gold-assisted method on a SiO_2_/Si substrate. The reference peak positions corresponding to free-standing monolayer MoS_2_ were adopted from literature data*^1^*. Based on the observed shifts of the Raman modes, slight compressive strain and n-type doping were identified within the monolayer.*

**Supporting Information 2: Raman and photoluminescence spectra of MoS_2_ and WSe_2_**

***Figure S2. Raman and photoluminescence spectra of MoS_2_ and WSe_2_. a)*** *Comparison of representative Raman signal of WSe_2_ bilayer and all measured spectra of monolayer showcasing the absence of* $B_{2g}$ *peak in the monolayer spectrum.* ***b)*** *PL spectrum of monolayer WSe_2_ showing A exciton peak position.* ***c)*** *Single Raman spectrum of MoS_2_ monolayers with marked signature* $E_{2g}^{1}$ *and* $A_{1g}$ *peaks.* ***d)*** *PL spectrum of monolayer MoS_2_ showcasing A exciton peak position.*


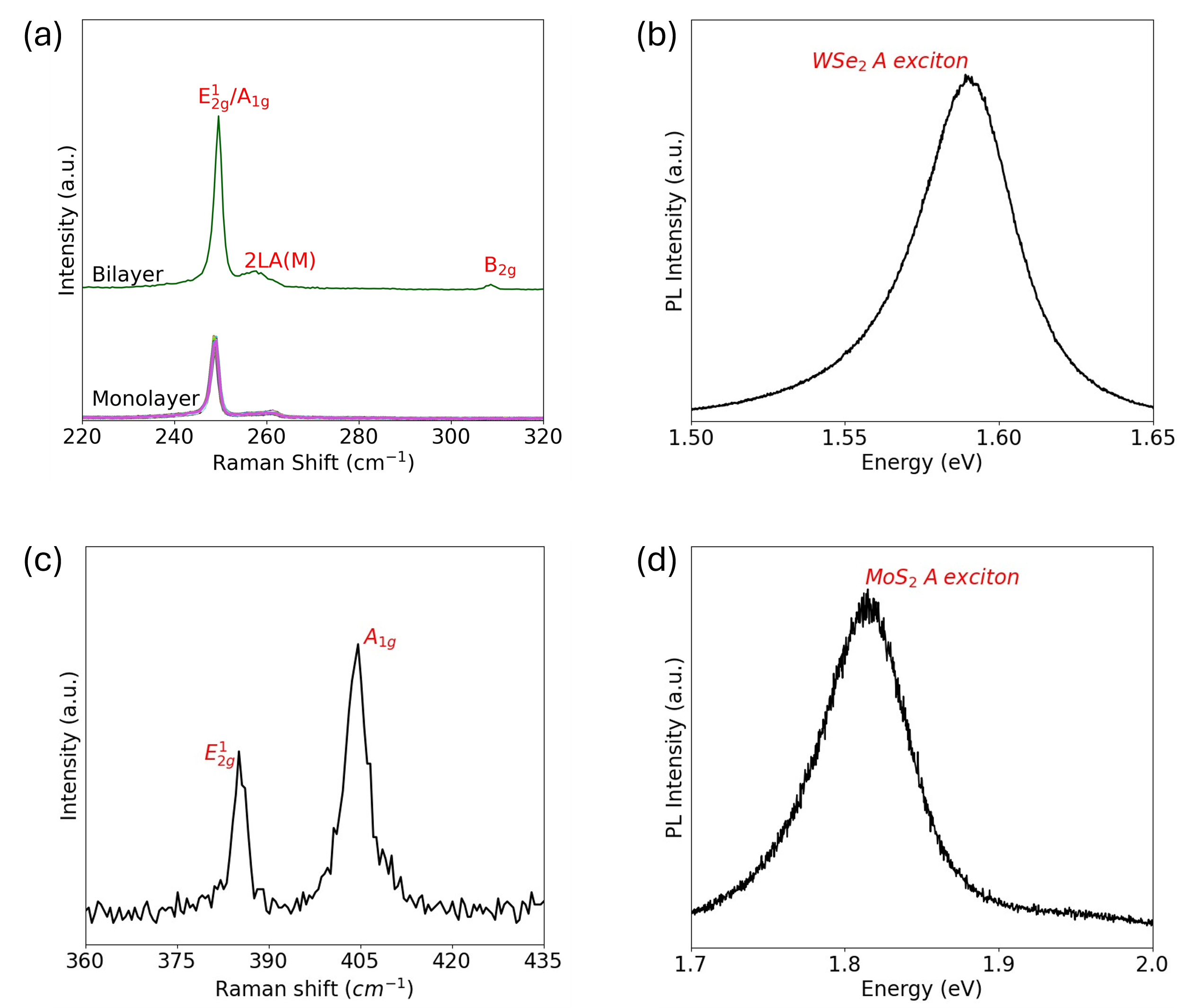


**Supporting Information 3: Optical image of exfoliated monolayers and fabricated FETs**

***Figure S3****.* ***Optical image of MoS_2_ and WSe_2_ at different fabrication stages.*** *(a) Contrast enhanced optical image of large-area monolayer of MoS_2_, where the flake marked as monolayer was exfoliated on an array of pre-fabricated gate islands using gold-assisted exfoliation. (b) Optical image of the same MoS_2_ region after device fabrication, which consists of an etching step to create the channel followed by contacts deposition. (c) Optical image of five MoS_2_ devices on a single back gate island. (d) Contrast enhanced optical image of monolayer WSe_2_ on local back gates. (e) Optical image of the same WSe_2_ region after device fabrication. (f) Optical image of a five WSe_2_ devices on a single back gate island.*


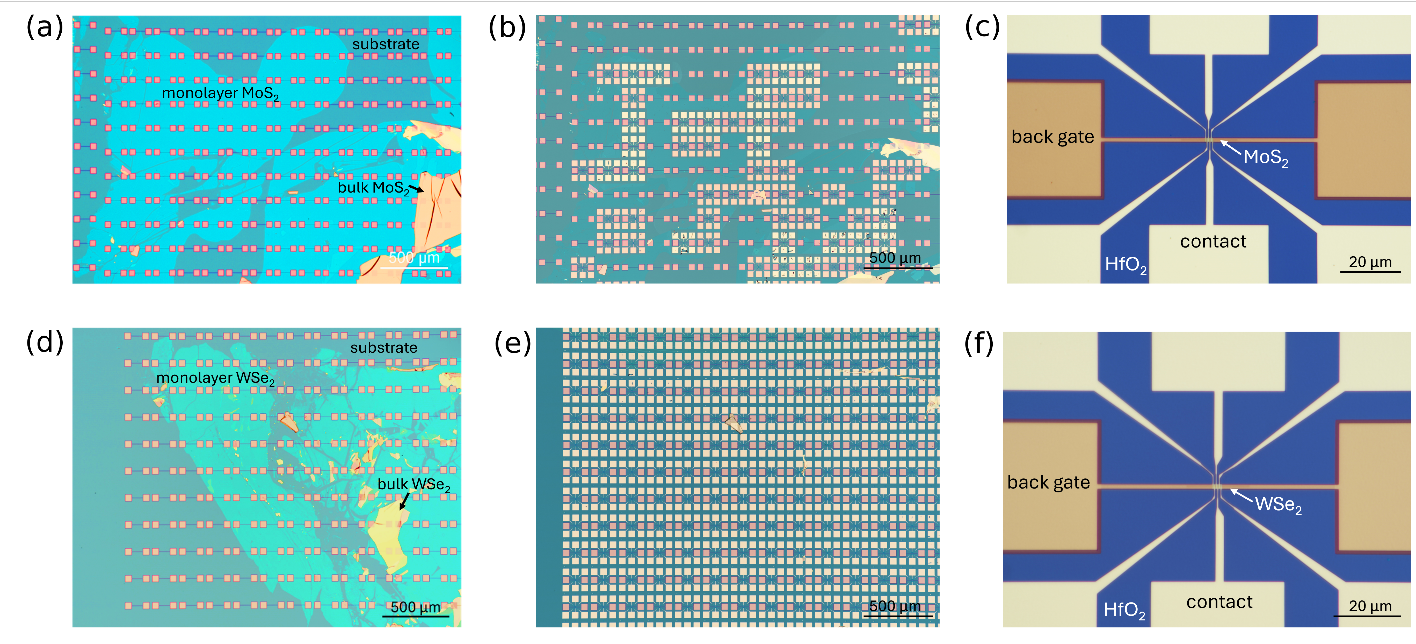


**Supporting Information 4: Leakage current**

***Figure S4. Gate leakage current in p-FET and n-FET devices.*** *Gate leakage current (*$I_{G}$*) in* ***a****) p-FET and* ***b)*** *n-FET devices measured for different* $V_{DS}$*. In both cases,* $I_{G}$ *remains at the pA/µm level and is independent of* $V_{DS}$ *and* $V_{BG}$*.*


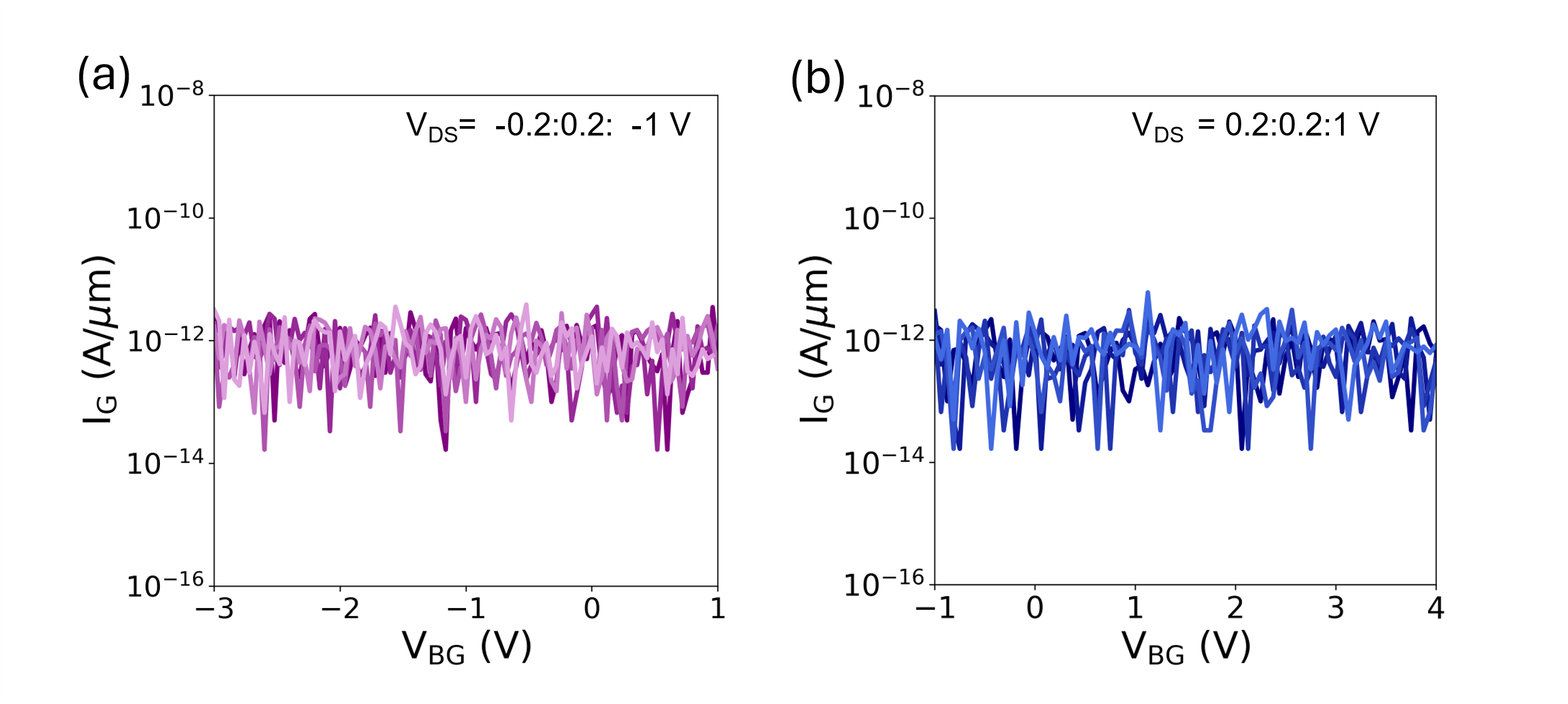


**Table S1: Benchmarking Table for MoS_2_ n-FETs**

| **Material** | **Synthesis** | **Contact** | **Dielectric** | $\mathbf{L}_{\mathbf{CH}}\boldsymbol{(}\mathbf{nm}\boldsymbol{)}$ | $\mathbf{I}_{\mathbf{ON}}\left( \frac{\boldsymbol{\mu A}}{\boldsymbol{\mu m}} \right)$ | $\mathbf{SS (mV/dec)}$ | $\frac{\mathbf{I}_{\mathbf{ON}}}{\mathbf{I}_{\mathbf{OFF}}}$ | **Ref.** |
| --- | --- | --- | --- | --- | --- | --- | --- | --- |
| 1L-MoS_2_ | CVD | Ni | 90nm SiO_2_ | 600 | 0.1 | 3500 | $10^{4}$ | ^2^ |
| 3L-MoS_2_ |  |  |  | 600 | 1 | 4000 |  |  |
| 1L-MoS_2_ | MOCVD | Bi | 100nm SiN_x_ | 120 | 560 | - | $10^{7}$ | ^3^ |
|  |  | Ni | 300nm SiO_2_ | 1000 | 2 | - | $10^{6}$ |  |
| 1L-MoS_2_ | CVD | Bi | 100nm SiN_x_ | 35 | 800 | 1670 | $10^{6}$ |  |
| 1L-MoS_2_ | CVD | Bi | 300nm SiO_2_ | 500 | 0.2 | - | - |  |
| 1L-MoS_2_ | CVD | Au | 30nm SiO_2_ | - | 300 | 1500 | $>10^{6}$ | ^4^ |
| 1L-MoS_2_ | MBE | Au | 5nm HfO_2_ | 25 | 92 | 128 | $10^{6}$ | ^5^ |
| 1L-MoS_2_ | MOCVD | Au | 10nm HfO_2_ | 500 | 120 | 2500 | $10^{8}$ | ^6^ |
| 1L-MoS_2_ | CVD | Sb | 100nm SiN_x_ | 100 | 495 | - | $10^{7}$ | ^7^ |
| 1L-MoS_2_ | CVD | Ni | 20nm HfO_2_ | 50 | 192 | 205 | $10^{8}$ | ^8^ |
| 1L-MoS_2_ | gold-assisted exfoliation | Au | 300nm SiO_2_ | 1500 | 0.1 | 100 | ${10}^{7}$ | ^9^ |
| 1L-MoS_2_ | CVD | Bi | 100 nm SiN_x_ | 100 | 544 | - | $10^{7}$ | ^7^ |
| 1L-MoS_2_ | MOCVD | Ni | 9nm Al_2_O_3_ /3nm HfO_2_/ 3nm Al_2_O_3_ | 300 | 33 | 79 | - | ^10^ |
| Multilayer MoS_2_ | scotch tape exfoliation | Ti/Au | 30nm Al_2_O_3_ | 1500 | 1 | 190 | ${10}^{5}$ | ^11^ |
| 1-15 layers MoS_2_ | scotch tape exfoliation | Au | 90nm SiO_2_ | 40 | 60 | - | - | ^12^ |
| **1L-MoS_2_** | **gold-assisted exfoliation** | **Au/Ti/Pt** | **10nm HfO_2_** | **200** | **63** | **102** | $\boldsymbol{10}^{\boldsymbol{8}}$ | **This work** |

**Table S2: Benchmarking Table for WSe_2_ p-FET**

| **Material** | **Synthesis** | **Contact** | **Dielectric** | **L_CH_ (nm)** | **I_ON_**  **(µA/µm)** | $\mathbf{SS (mV/dec)}$ | $\frac{\mathbf{I}_{\mathbf{ON}}}{\mathbf{I}_{\mathbf{OFF}}}$ | **Dopant** | **Ref.** |
| --- | --- | --- | --- | --- | --- | --- | --- | --- | --- |
| 1L-WSe_2_ | CVT | Pd | 17.5 nm ZrO_2_ | 9400 | 10 | 60 | 10^6^ | NO_2_ | ^13^ |
| Multilayer-WSe_2_ | CVT | Pd/Pt | 30 nm SiO_2_ | 400 | 100 | - | 10 | MoO_3_ | ^14^ |
| 1L-WSe_2_ | CVD | Ti/Pd/Ni | 2.8 nm HfO_2_ | 65 | 300 | 225 | ~2×10^6^ | NO_X_ | ^15^ |
| 1L-WSe_2_ | CVD | Cr/Au | SiO_2_ | 1800 | 17.9 | 4000 | 2.2×10^7^ | Nb | ^16^ |
| 2L-WSe_2_ | CVT | Ti/Pt | SiO_2_ | **-** | 7 | ~ 100 | 2×10^8^ | WO_3_ | ^17^ |
| 1L-WSe_2_ | CVD | Ni/Au | 120 nm SiO_2_ | 3000 | 10 | > 3000 | 10^6^ | Nb | ^18^ |
| 1L-WSe_2_ | MOCVD | Pd | 9nm Al_2_O_3_ /3nm HfO_2_/ 3nm Al_2_O_3_ | 300 | 16 | 244 | **-** | **-** | ^19^ |
| 1L-WSe_2_ | CVD | Ti/Pt/Au | 6 nm HfO_2_ | 55 | 546 | 75 | 10^9^ | NO | ^20^ |
| multilayer WSe_2_ | MOCVD | Ru | 10 nm HfO_2_ | 140 | 200 | 7500 | 2x10^6^ | **-** | ^21^ |
| 1L-WSe_2_ | MOCVD | Ru | 4.6 nm HfO_2_ | 50 | 92 | 180 | 10^8^ | - | ^22^ |
| 2L-WSe_2_ | MOCVD | Pd | 25 nm Al_2_O_3_ | 20 | 40 | 630 | >10^5^ | WO_x_Se_y_ layer | ^23^ |
| 1L-WSe_2_ | MOCVD | Pd | 9nm Al_2_O_3_ /3nm HfO_2_/ 3nm Al_2_O_3_ | 300 | 10 | 450 | - | - | ^10^ |
| ~5L-WSe_2_ | Scotch tape exfoliation | Pt/Pd | 8 nm of HfO_2_ | 400 | 97 | 140 | 10^8^ |  | ^24^ |
| multilayers WSe_2_ | Scotch tape exfoliation | Pd/Au | 285 nm SiO_2_ | 3.5 | 20 | 320 | 10^8^ | - | ^25^ |
| **1L-WSe_2_** | **gold-assisted exfoliation** | **Pd/Pt** | **10nm HfO2** | **200** | **88** | **85** | $\mathbf{10}^{\mathbf{7}}$ | **NO** | **This work** |

**Supporting Information 5: Output signal of a CMOS inverter**

***Figure S5.*** *A single cycle of the output signal* $V_{OUT}$ *of a representative CMOS inverter, acquired in response to a square-wave input signal switching between GND and a* $V_{DD}$ *of 3 V.*


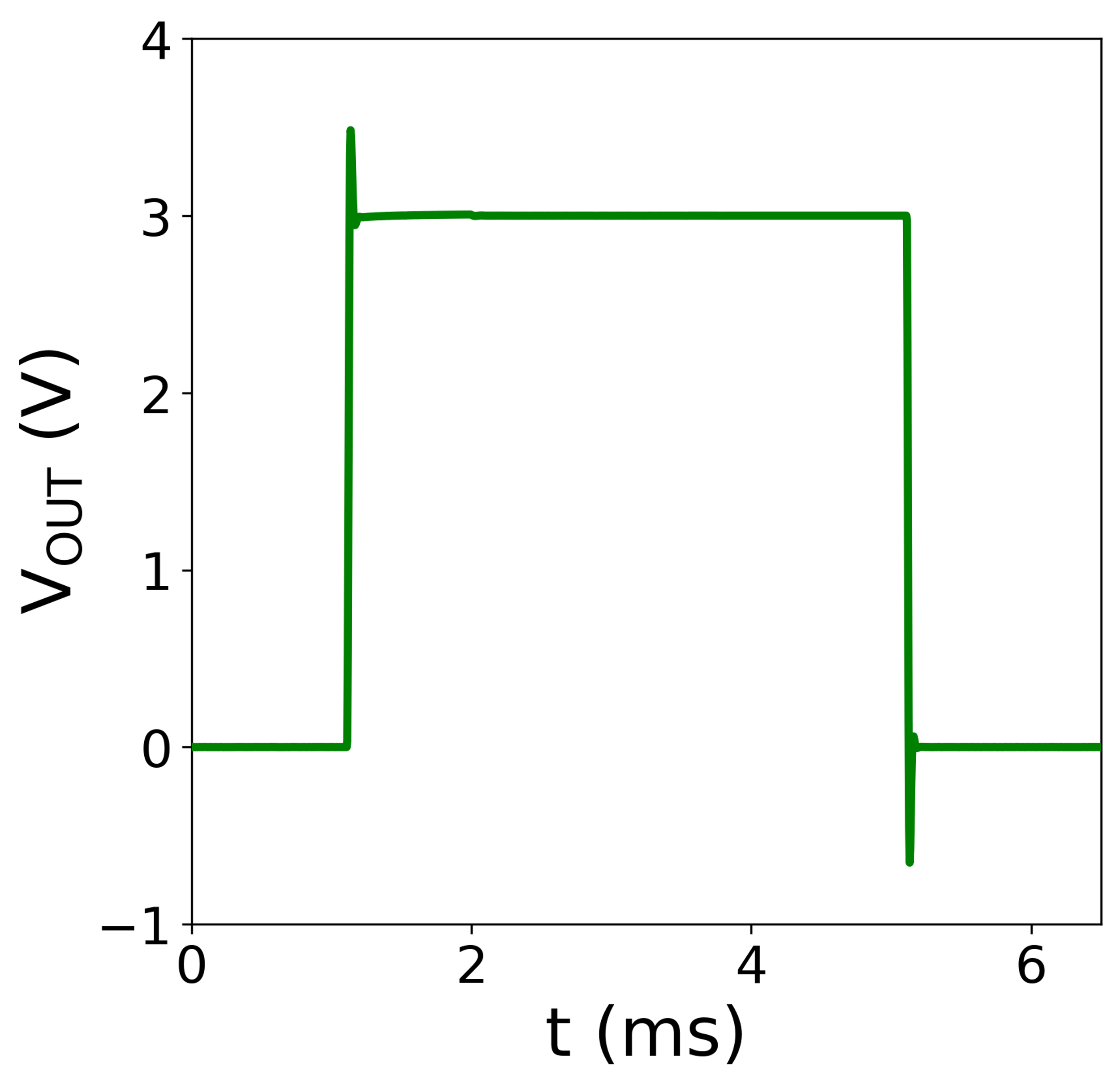


**Supporting Information 6: Schematic illustration of gold-assisted exfoliation.**

***Figure S6. Gold-assisted exfoliation.*** *Schematic of the exfoliation method using a TRT/PMMA/Au stack referred to as gold tape. The gold tape is first pressed onto a bulk crystal to pick up a monolayer, then pressed against the target substrate. Upon removing the tape, the monolayer remains adhered to the substrate.*


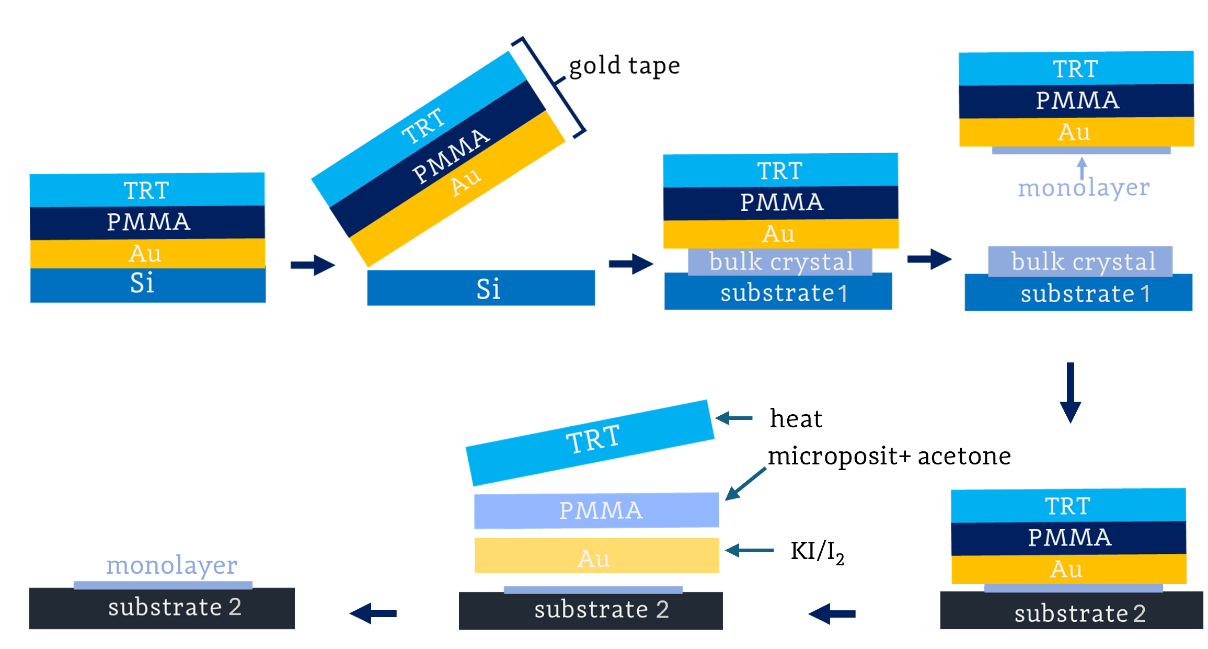


REFERENCES

(1) Panasci, S. E.; Schilirò, E.; Greco, G.; Cannas, M.; Gelardi, F. M.; Agnello, S.; Roccaforte, F.; Giannazzo, F. Strain, Doping, and Electronic Transport of Large Area Monolayer MoS _2_ Exfoliated on Gold and Transferred to an Insulating Substrate. *ACS Appl. Mater. Interfaces* **2021**, *13* (26), 31248–31259. https://doi.org/10.1021/acsami.1c05185.

(2) Cheng, Z.; Yu, Y.; Singh, S.; Price, K.; Noyce, S. G.; Lin, Y.-C.; Cao, L.; Franklin, A. D. Immunity to Contact Scaling in MoS_2_ Transistors Using in Situ Edge Contacts. *Nano Lett.* **2019**, *19* (8), 5077–5085. https://doi.org/10.1021/acs.nanolett.9b01355.

(3) Shen, P.-C.; Su, C.; Lin, Y.; Chou, A.-S.; Cheng, C.-C.; Park, J.-H.; Chiu, M.-H.; Lu, A.-Y.; Tang, H.-L.; Tavakoli, M. M.; Pitner, G.; Ji, X.; Cai, Z.; Mao, N.; Wang, J.; Tung, V.; Li, J.; Bokor, J.; Zettl, A.; Wu, C.-I.; Palacios, T.; Li, L.-J.; Kong, J. Ultralow Contact Resistance between Semimetal and Monolayer Semiconductors. *Nature* **2021**, *593* (7858), 211–217. https://doi.org/10.1038/s41586-021-03472-9.

(4) McClellan, C. J.; Yalon, E.; Smithe, K. K. H.; Suryavanshi, S. V.; Pop, E. High Current Density in Monolayer MoS_2_ Doped by AlO *_x_* . *ACS Nano* **2021**, *15* (1), 1587–1596. https://doi.org/10.1021/acsnano.0c09078.

(5) Dorow, C. J.; Penumatcha, A.; Kitamura, A.; Rogan, C.; O’Brien, K. P.; Lee, S.; Ramamurthy, R.; Cheng, C.-Y.; Maxey, K.; Zhong, T.; Tronic, T.; Holybee, B.; Richards, J.; Oni, A.; Lin, C.-C.; Naylor, C. H.; Arefin, N.; Metz, M.; Bristol, R.; Clendenning, S. B.; Avci, U. Gate Length Scaling beyond Si: Mono-Layer 2D Channel FETs Robust to Short Channel Effects. In *2022 International Electron Devices Meeting (IEDM)*; IEEE: San Francisco, CA, USA, 2022; p 7.5.1-7.5.4. https://doi.org/10.1109/IEDM45625.2022.10019524.

(6) Kwon, J.; Seol, M.; Yoo, J.; Ryu, H.; Ko, D.-S.; Lee, M.-H.; Lee, E. K.; Yoo, M. S.; Lee, G.-H.; Shin, H.-J.; Kim, J.; Byun, K.-E. 200-Mm-Wafer-Scale Integration of Polycrystalline Molybdenum Disulfide Transistors. *Nat Electron* **2024**, *7* (5), 356–364. https://doi.org/10.1038/s41928-024-01158-4.

(7) Chou, A.-S.; Wu, T.; Cheng, C.-C.; Zhan, S.-S.; Ni, I.-C.; Wang, S.-Y.; Chang, Y.-C.; Liew, S.-L.; Chen, E.; Chang, W.-H.; Wu, C.-I.; Cai, J.; Wong, H.-S. P.; Wang, H. Antimony Semimetal Contact with Enhanced Thermal Stability for High Performance 2D Electronics. In *2021 IEEE International Electron Devices Meeting (IEDM)*; IEEE: San Francisco, CA, USA, 2021; p 7.2.1-7.2.4. https://doi.org/10.1109/IEDM19574.2021.9720608.

(8) Chou, A.-S.; Shen, P.-C.; Cheng, C.-C.; Lu, L.-S.; Chueh, W.-C.; Li, M.-Y.; Pitner, G.; Chang, W.-H.; Wu, C.-I.; Kong, J.; Li, L.-J.; Wong, H. S. P. High On-Current 2D nFET of 390 μA/Μm at VDS = 1V Using Monolayer CVD MoS2 without Intentional Doping. In *2020 IEEE Symposium on VLSI Technology*; IEEE: Honolulu, HI, USA, 2020; pp 1–2. https://doi.org/10.1109/VLSITechnology18217.2020.9265040.

(9) Huang, Y.; Pan, Y.-H.; Yang, R.; Bao, L.-H.; Meng, L.; Luo, H.-L.; Cai, Y.-Q.; Liu, G.-D.; Zhao, W.-J.; Zhou, Z.; Wu, L.-M.; Zhu, Z.-L.; Huang, M.; Liu, L.-W.; Liu, L.; Cheng, P.; Wu, K.-H.; Tian, S.-B.; Gu, C.-Z.; Shi, Y.-G.; Guo, Y.-F.; Cheng, Z. G.; Hu, J.-P.; Zhao, L.; Yang, G.-H.; Sutter, E.; Sutter, P.; Wang, Y.-L.; Ji, W.; Zhou, X.-J.; Gao, H.-J. Universal Mechanical Exfoliation of Large-Area 2D Crystals. *Nat Commun* **2020**, *11* (1), 2453. https://doi.org/10.1038/s41467-020-16266-w.

(10) Jayachandran, D.; Pendurthi, R.; Sadaf, M. U. K.; Sakib, N. U.; Pannone, A.; Chen, C.; Han, Y.; Trainor, N.; Kumari, S.; Mc Knight, T. V.; Redwing, J. M.; Yang, Y.; Das, S. Three-Dimensional Integration of Two-Dimensional Field-Effect Transistors. *Nature* **2024**, *625* (7994), 276–281. https://doi.org/10.1038/s41586-023-06860-5.

(11) Sik Hwang, W.; Remskar, M.; Yan, R.; Kosel, T.; Kyung Park, J.; Jin Cho, B.; Haensch, W.; (Grace) Xing, H.; Seabaugh, A.; Jena, D. Comparative Study of Chemically Synthesized and Exfoliated Multilayer MoS2 Field-Effect Transistors. *Applied Physics Letters* **2013**, *102* (4), 043116. https://doi.org/10.1063/1.4789975.

(12) English, C. D.; Shine, G.; Dorgan, V. E.; Saraswat, K. C.; Pop, E. Improved Contacts to MoS _2_ Transistors by Ultra-High Vacuum Metal Deposition. *Nano Lett.* **2016**, *16* (6), 3824–3830. https://doi.org/10.1021/acs.nanolett.6b01309.

(13) Fang, H.; Chuang, S.; Chang, T. C.; Takei, K.; Takahashi, T.; Javey, A. High-Performance Single Layered WSe_2_ p-FETs with Chemically Doped Contacts. *Nano Lett.* **2012**, *12* (7), 3788–3792. https://doi.org/10.1021/nl301702r.

(14) Cai, L.; McClellan, C. J.; Koh, A. L.; Li, H.; Yalon, E.; Pop, E.; Zheng, X. Rapid Flame Synthesis of Atomically Thin MoO_3_ down to Monolayer Thickness for Effective Hole Doping of WSe_2_. *Nano Lett.* **2017**, *17* (6), 3854–3861. https://doi.org/10.1021/acs.nanolett.7b01322.

(15) Chiang, C.-C.; Lan, H.-Y.; Pang, C.-S.; Appenzeller, J.; Chen, Z. Air-Stable P-Doping in Record High-Performance Monolayer WSe_2_ Devices. *IEEE Electron Device Lett.* **2022**, *43* (2), 319–322. https://doi.org/10.1109/LED.2021.3135312.

(16) Vu, V. T.; Vu, T. T. H.; Phan, T. L.; Kang, W. T.; Kim, Y. R.; Tran, M. D.; Nguyen, H. T. T.; Lee, Y. H.; Yu, W. J. One-Step Synthesis of NbSe_2_ /Nb-Doped-WSe_2_ Metal/Doped-Semiconductor van Der Waals Heterostructures for Doping Controlled Ohmic Contact. *ACS Nano* **2021**, *15* (8), 13031–13040. https://doi.org/10.1021/acsnano.1c02038.

(17) Yang, S.; Lee, G.; Kim, J. Selective P-Doping of 2D WSe_2_ *via* UV/Ozone Treatments and Its Application in Field-Effect Transistors. *ACS Appl. Mater. Interfaces* **2021**, *13* (1), 955–961. https://doi.org/10.1021/acsami.0c19712.

(18) Pandey, S. K.; Alsalman, H.; Azadani, J. G.; Izquierdo, N.; Low, T.; Campbell, S. A. Controlled P-Type Substitutional Doping in Large-Area Monolayer WSe_2_ Crystals Grown by Chemical Vapor Deposition. *Nanoscale* **2018**, *10* (45), 21374–21385. https://doi.org/10.1039/C8NR07070A.

(19) Pendurthi, R.; Sakib, N. U.; Sadaf, M. U. K.; Zhang, Z.; Sun, Y.; Chen, C.; Jayachandran, D.; Oberoi, A.; Ghosh, S.; Kumari, S.; Stepanoff, S. P.; Somvanshi, D.; Yang, Y.; Redwing, J. M.; Wolfe, D. E.; Das, S. Monolithic Three-Dimensional Integration of Complementary Two-Dimensional Field-Effect Transistors. *Nat. Nanotechnol.* **2024**, *19* (7), 970–977. https://doi.org/10.1038/s41565-024-01705-2.

(20) Lan, H.-Y.; Tripathi, R.; Liu, X.; Appenzeller, J.; Chen, Z. Wafer-Scale CVD Monolayer WSe_2_ p-FETs with Record-High 727 μA/Μm I_on_ and 490 μS/ Μm g_max_ via Hybrid Charge Transfer and Molecular Doping. In *2023 International Electron Devices Meeting (IEDM)*; IEEE: San Francisco, CA, USA, 2023; pp 1–4. https://doi.org/10.1109/IEDM45741.2023.10413736.

(21) Dorow, C. J.; Schram, T.; Smets, Q.; O’Brien, K. P.; Maxey, K.; Lin, C.-C.; Panarella, L.; Kaczer, B.; Arefin, N.; Roy, A.; Jordan, R.; Oni, A.; Penumatcha, A.; Naylor, C. H.; Kavrik, M.; Cott, D.; Graven, B.; Afanasiev, V.; Morin, P.; Asselberghs, I.; Lockhart De La Rosa, C. J.; Sankar Kar, G.; Metz, M.; Avci, U. Exploring Manufacturability of Novel 2D Channel Materials: 300 Mm Wafer-Scale 2D NMOS & PMOS Using MoS_2_ , WS_2_ , & WSe_2_. In *2023 International Electron Devices Meeting (IEDM)*; IEEE: San Francisco, CA, USA, 2023; pp 1–4. https://doi.org/10.1109/IEDM45741.2023.10413874.

(22) Mortelmans, W.; Buragohain, P.; Rogan, C.; Kitamura, A.; Dorow, C. J.; O’Brien, K. P.; Ramamurthy, R.; Lux, J.; Zhong, T.; Harlson, S.; Gillispie, E.; Wilson, T.; Oni, A.; Penumatcha, A.; Kavrik, M.; Maxey, K.; Kozhakhmetov, A.; Lin, C.-C.; Lee, S.; Vyatskikh, A.; Arefin, N.; Fischer, P.; Kevek, J.; Tronic, T.; Metz, M.; Clendenning, S.; Avci, U. Record Performance in GAA 2D NMOS and PMOS Using Monolayer MoS2 and WSe2 with Scaled Contact and Gate Length. In *2024 IEEE Symposium on VLSI Technology and Circuits (VLSI Technology and Circuits)*; IEEE: Honolulu, HI, USA, 2024; pp 1–2. https://doi.org/10.1109/VLSITechnologyandCir46783.2024.10631395.

(23) Oberoi, A.; Han, Y.; Stepanoff, S. P.; Pannone, A.; Sun, Y.; Lin, Y.-C.; Chen, C.; Shallenberger, J. R.; Zhou, D.; Terrones, M.; Redwing, J. M.; Robinson, J. A.; Wolfe, D. E.; Yang, Y.; Das, S. Toward High-Performance p-Type Two-Dimensional Field Effect Transistors: Contact Engineering, Scaling, and Doping. *ACS Nano* **2023**, *17* (20), 19709–19723. https://doi.org/10.1021/acsnano.3c03060.

(24) Patoary, N. H.; Xie, J.; Zhou, G.; Al Mamun, F.; Sayyad, M.; Tongay, S.; Esqueda, I. S. Improvements in 2D P-Type WSe2 Transistors towards Ultimate CMOS Scaling. *Sci Rep* **2023**, *13* (1), 3304. https://doi.org/10.1038/s41598-023-30317-4.

(25) Liu, X.; Pan, Y.; Yang, J.; Qu, D.; Li, H.; Yoo, W. J.; Sun, J. High Performance WSe2 P-MOSFET with Intrinsic n-Channel Based on Back-to-Back p–n Junctions. *Applied Physics Letters* **2021**, *118* (23), 233101. https://doi.org/10.1063/5.0036343.
